# Endolysin, a Promising Solution against Antimicrobial Resistance

**DOI:** 10.3390/antibiotics10111277

**Published:** 2021-10-20

**Authors:** Mujeeb ur Rahman, Weixiao Wang, Qingqing Sun, Junaid Ali Shah, Chao Li, Yanmei Sun, Yuanrui Li, Bailing Zhang, Wei Chen, Shiwei Wang

**Affiliations:** 1 Key Laboratory of Resources Biology and Biotechnology in Western China, Ministry of Education, College of Life Sciences, Northwest University, Xi’an 710069, China; mujeeb@stumail.nwu.edu.cn (M.u.R.); sunqingqingls@163.com (Q.S.); lichaoxb@163.com (C.L.); sunyanmei@nwu.edu.cn (Y.S.); liyr13579@126.com (Y.L.); 2 Clinical Research Center, The Second Hospital of Nanjing, Nanjing University of Chinese Medicine, Nanjing 210003, China; wangweixiao2021@njucm.edu.cn; 3 College of Life Sciences, Jilin University, Changchun 130012, China; Junaid1316@jlu.edu.cn; 4 Department of Laboratory Medicine, The Second Affiliated Hospital of Nanchang University, Nanchang 330006, China; zbailing1974@126.com

**Keywords:** antimicrobial resistance, endolysin, biofilm, food safety, pathogen detection

## Abstract

Antimicrobial resistance (AMR) is a global crisis for human public health which threatens the effective prevention and control of ever-increasing infectious diseases. The advent of pandrug-resistant bacteria makes most, if not all, available antibiotics invalid. Meanwhile, the pipeline of novel antibiotics development stagnates, which prompts scientists and pharmacists to develop unconventional antimicrobials. Bacteriophage-derived endolysins are cell wall hydrolases which could hydrolyze the peptidoglycan layer from within and outside of bacterial pathogens. With high specificity, rapid action, high efficiency, and low risk of resistance development, endolysins are believed to be among the best alternative therapeutic agents to treat multidrug resistant (MDR) bacteria. As of now, endolysins have been applied to diverse aspects. In this review, we comprehensively introduce the structures and activities of endolysins and summarize the latest application progress of recombinant endolysins in the fields of medical treatment, pathogen diagnosis, food safety, and agriculture.

## 1. Introduction

Humans have used antibiotics for more than half a century to counter infectious diseases caused by pathogenic bacteria. Overuse and misuse of antibiotics have contributed to a rise in the number of antibiotic-resistant strains, including multidrug-resistant (MDR), extensively drug-resistant (XDR), and even pandrug-resistant (PDR) strains. The inherent heredity and physiology transmitted vertically within species, and the tendency of bacteria to exchange various genes horizontally between species and genera, have been recommended as possible causes of resistance to antibiotics [1,2,3]. Due to the occurrence of incurable infections, the number of medical procedure failures is expected to increase in the near future. As well, antibiotic-resistant strains are also responsible for the increased cost of livestock breeding, food industry, and agriculture. In this “post-antibiotic era”, it is imperative to search for new therapeutic approaches in the battle against bacterial infections. 

Bacteriophages (phages) are the natural enemies of bacteria, which were first used as therapeutic agents in humans in 1919, just a few years after they were discovered [4]. Phages have a range of potential benefits compared to antibiotics. The major advantage of phages is their specificity for target bacteria, which significantly reduces the damage to the host’s normal flora. The phages are self-limiting, i.e., they need their hosts for continuous growth. If the specific hosts are not available, they will not last long [5]. However, the human immune system eliminates incoming phages, posing an obstacle to their use as a therapeutic agent [6,7]. Especially, the strong antibody response of in vivo phage therapy causes phages to be cleared more quickly, making long-term use of phages impossible [5]. Another disadvantage of phages is their narrow host range, making it difficult to look for suitably paired phages for a given bacterial pathogen. At last, it is very important to ensure that phage preparations are free of bacteria and bacterial toxins during the preparation of phage stocks, which increases the production cost and technical challenge. Thus, rather than administering the whole virions, one choice is to use the phage lytic enzyme, endolysin [8,9,10].

Endolysins are hydrolases produced by phages which function in vivo to lyse bacterial cell walls and release progeny phages at the end of a replication cycle [11]. These enzymes are remarkably efficient in hydrolyzing the peptidoglycan layer, resulting in a sudden drop in turgor pressure and osmotic lysis to cause bacterial cell death [12]. Endolysins are considered as a promising class of antibiotics derived from enzymes known as “enzybiotics”. The major advantage of endolysins over conventional broad-spectrum antibiotics is their high specificity. Endolysins exhibit specific bactericidal activity and do not kill the beneficial microbiota [13,14]. Through molecular engineering, the lytic spectrum of an endolysin could be changed [15]. Meanwhile, endolysins have other advantages, such as rapid bacterial cell lysis, a low risk of resistance, synergistic activity with different antibacterial agents, and the ability to effectively function in biofilms and on mucosal surfaces [16,17,18,19,20]. Due to these unique properties, endolysins are highly ranked alternatives in eradicating drug-resistant pathogens [13,21,22,23]. In the last decade, recombinant endolysins have been applied in many fields to combat MDR bacteria [13,20,24,25]. This review aims to introduce the diverse protein architectures of different endolysins and comprehensively summarize the progress of endolysin application in medical practice, pathogen diagnosis, food safety, and agriculture. Moreover, the commercialization of endolysins from lab to market is discussed.

## 2. Architecture of Endolysins

Phage endolysins are analogous to bacterial lysins in structure and function, which are closely linked to the small family of mammalian peptidoglycan recognition proteins [26]. The endolysin architectures are different between Gram-positive and Gram-negative phages. As for Gram-positive phages, most endolysins are composed of two domains, an N-terminal enzymatic activity domain (EAD) and a C-terminal cell wall binding domain (CBD), which are connected by a short flexible linker [27]. EAD contributes to the cleavage of different bonds in peptidoglycan, while CBD recognizes and binds specifically to the receptor of bacterial cell walls [28]. Besides this architecture, some endolysins contain unusual structures. For example, staphylococcal endolysin, λSA2, has a central CBD and two flanking EADs [29]. Recently, a novel *Bacillus cereus* endolysin, LysPBC2, was confirmed to have an extra spore binding domain (SBD), besides EAD and CBD, which specifically binds *B. cereus* spores but not to its vegetative cells [30]. *Streptococcus dysgalactiae* phage endolysin PlySK1249 was composed of an EAD, a central CBD, and a C-terminal CHAP domain, which is a cysteine, histidine-dependent amidohydrolases/peptidase. Interestingly, the CHAP domain of PlySK1249 was a nonbacteriolytic endopeptidase, which acted as a dechaining enzyme and exhibited a synergistic effect with the lytic amidase domain for peptidoglycan digestion and bacteriolysis [31]. Remarkably, the modular structure of Gram-positive phage endolysins facilitates protein engineering to modify bacteriolytic activity, specificity, solubility, and other physicochemical properties of endolysins [32]. For example, the full-length or EAD of LysB4, an endolysin from the *B. cereus* phage B4, was fused to LysSA11, an endolysin from *S. aureus* phage SA11, to form a hybrid endolysin and simultaneously control *Staphylococcus aureus* and *B. cereus* [33]. *S. aureus* phage Twort endolysin (PlyTW) was composed of three domains, including a CHAP domain, an amidase-2 domain, and a CBD. Deletion of the amidase-2 domain formed a novel shorter endolysin with stronger activity [34].

In contrast, most endolysins of Gram-negative phages are small single-domain globular proteins with molecular mass between 15 and 20 kDa, usually without a specific CBD [14]. Exceptionally, a few Gram-negative endolysins have a modular structure, such as *Pseudomonas* endolysin KZ144, *P. fluorescens* phage OBP endolysin OBPgp279, and *Burkholderia* phage AP3 endolysin AP3gp15 [17,35,36]. The modular endolysins with a Gram-negative background display a unique property with an N-terminal CBD and a C-terminal EAD [36]. Interestingly, CBDs from these endolysins demonstrate a broad binding spectrum, which is different from Gram-positive endolysins [28]. 

Most endolysins are the product of a single gene. However, one of the endolysins against *Streptococcus* spp., PlyC, is encoded by *plyCA* and *plyCB*. PlyC is a multimeric endolysin from the streptococcal C1 phage, comprising of two components. PlyCA is essential for enzymatic activity and PlyCB is able to direct streptococcal cell-wall-specific binding [37]. X-ray crystal diffraction revealed that the PlyC structure consists of an EAD (PlyCA) and eight copies of CBDs (PlyCB) [38]. Additionally, a few multimeric endolysins encoded by a single gene have been detected, such as CD27L from *Clostridium difficile* phage, CTP1L from *C. tyrobutyricum* phage [39,40], and Lys170 from enterococcal phage F170/08 [41]. The full-length endolysin and its CBD fragment are expressed in phage F170/08, respectively, and they interact to form the fully active endolysin. The CBD part is produced from an in-frame, alternative translation start site of the same gene [41].

## 3. Antimicrobial Activity of Endolysins

Depending on the mode of action, EADs are categorized into three groups: (a) glycosidases, cleaving the glycan portion of peptidoglycan (MurNAc-GlcNAc); (b) amidases, cleaving the amide bond between the glycan moiety (MurNAc) and the peptide moiety (L-alanine); and (c) endopeptidase, cleaving the peptide bond between two amino acids of the stem peptide [25]. It is worthy to note that CHAP is a special case, as this type is not classified based on which bond in peptidoglycan they cleave, but on their catalytic mechanism [42]. CHAP domains have an invariant cysteine and histidine residue in their active site for substrate cleavage [43]. The enzymatic activity of endolysins is influenced by the composition of cell walls. As for Gram-positive bacteria, the cell wall is comprised of an internal cytoplasmic cell membrane and a thick peptidoglycan layer [44]. When applied exogenously, endolysins readily access the peptidoglycan layer and hydrolyze the basic bonds of peptidoglycan, resulting in osmotic lysis and cell death [45]. However, the cell wall of Gram-negative bacteria comprises an internal cytoplasmic cell membrane, a peptidoglycan layer, and an outer membrane with a lipopolysaccharide layer [45], which inhibits access to the peptidoglycan layer [46]. 

**Figure 1:**
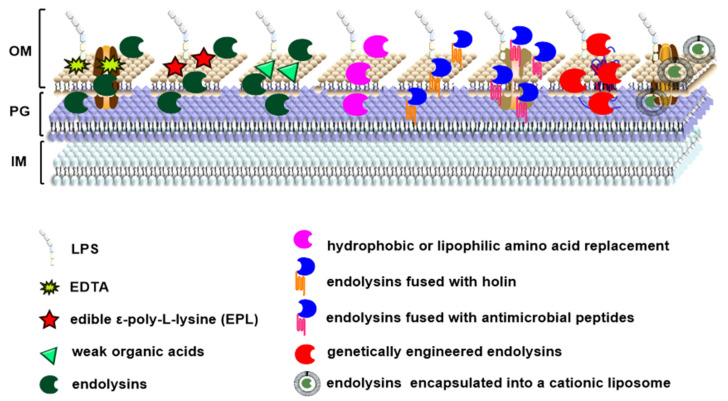
Different strategies of overcoming the barrier of Gram-negative bacterial outer membrane in the endolysin application.

There are a few strategies to overcome the disadvantages of endolysins from Gram-negative phages (Figure 1). At first, EDTA is often used to permeabilize the outer membrane. Endolysin LysSP1 from *Salmonella* phage SLMP1 displayed a very broad lytic spectrum against Gram-negative and Gram-positive bacteria with the help of 5 mM EDTA [47]. Similarly, the lytic activity of the endolysin LysPN09 from *P. syringae* pv. *actinidiae* phage PN09 was improved at the presence of 1 mM EDTA [48]. Secondly, the edible ε-poly-L-lysine (EPL) can be used as an outer-membrane permeabilizer for some endolysins targeting food-borne pathogens. *Salmonella* endolysin LyS15S6 lysed *Salmonella* and three species of *Enterobacteriaceae* with EPL at a very low concentration [49]. Thirdly, endolysins exhibit increased and broader antibacterial activity in the presence of weak organic acids. *Acinetobacter baumannii* phage endolysin ABgp46 rapidly killed *A. baumannii*, *P. aeruginosa*, and *Salmonella enterica* serovar Typhimurium strains with the help of 3.65 mM citric acid and 4.55 mM malic acid [50]. Fourthly, amino acid replacement in endolysin improves its antimicrobial activity. After 3–12 hydrophobic amino acids were successfully added to the C-terminus of *E. coli* phage endolysin Lysep3, the modified Lysep3 was able to kill *E. coli* from outside of the cells [51]. Fifthly, genetic engineering of endolysins has been applied to make innolysins and artilysins, which can pass through the outer membrane barrier and increase the lytic activity [11,52]. Recently, to treat *Helicobacter pylori* infection, holin and a section of polypeptides were fused to the endolysin of *H. pylori* to create a novel artilysin [53]. Holin is a transmembrane protein which punches holes in the cell membrane. Usually, polycations and hydrophobic polypeptides were chosen which enable penetration of the outer membrane [53]. Sixthly, endolysins are directly fused with antimicrobial peptides. The coliphage LysECD7 was fused with the N-terminus sheep myeloid peptide (SMAP) to form LysECD7-SAMP which extended the antimicrobial spectrum and enhanced the activity. This modified endolysin can be not only used for topical treatment but also for systemic applications in the bloodstream and tissues [54]. At last, endolysins can be delivered by encapsulation into a cationic liposome. For example, *Salmonella* phage endolysin BSP16Lys was encapsulated by a liposome comprised of dipalmitoylphosphatidylcholine, cholesterol, and hexadecylamine, which inhibited the growth of *S.* Typhimurium and *E. coli* [55].

To our interest, some Gram-negative endolysins possess the ability to lyse the bacteria even in the absence of membrane permeabilizer. PD-6A3 was a novel phage of *A. baumannii*, which could not only inhibit *A. baumannii* but also *E. coli* and methicillin-resistant *S. aureus* [56]. Similarly, another *A. baumannii* phage endolysin, LysAB54, showed high antibacterial activity against multiple Gram-negative pathogens [57]. Ts2631, an endolysin from extremophilic *Thermus scotoductus* phage, was able to lyse not only its host, but also *T. thermophilus*, *A. baumannii*, *P. aeruginosa*, and *C. freundii* without membrane permeabilizer [58]. Comparative study of the structures of these Gram-negative endolysins with or without the capacity of lysing bacteria on their own is needed to reveal this interesting phenomenon. 

The novel endolysins found in the PubMed database since 2019 are listed in Table 1 and Table 2.

## 4. Anti-Biofilm Activity of Endolysins

Biofilms are a major concern in food and clinical setting. They form in critical areas and cause contamination to threaten the effectiveness of the existing procedures during the food process [110]. Furthermore, antibiotic-resistant bacteria housed within the biofilm network lead to treatment failure in surgeries and chronic wounds [111]. Therefore, the development of novel anti-biofilm techniques has become essential in order to provide additional control strategies. 

Some anti-biofilm agents have been found, such as phages [112], metal oxide nanoparticles [113,114], and photosensitizers [115,116]. The most obvious disadvantage of phage and antibiotic therapy is that resistant bacteria are readily produced [117]. Although metal oxide nanoparticles exhibit miscellaneous functions, such as antibacterial agents, biosensors in drug and delivery formulations, and cancer therapy, some in vitro and in vivo studies have demonstrated that nanoparticles exposure can provoke oxidative stress, inflammatory responses, myocardial infarction, and thrombosis. Therefore, the cellular and molecular toxicology of nanoparticles should be investigated before use [113,118]. Photosensitizers have poor selectivity towards pathogens and also lead to the occurrence of resistant bacteria. In addition, the short excitation light wave of photosensitizers has poor penetrating power [116,119,120]. By contrast, endolysins do not produce resistant bacteria due to the importance and conservation of their targets, peptidoglycan layer, for the viability of bacteria and they also obtain access to pathogens in inner tissues and organs in vivo [112]. Therefore, endolysins are promising anti-biofilm agents that are capable of eliminating biofilms. 

One special biofilm issue with extensive attention is foreign body-associated infection (FBAI), which is due to bacterial adherence to and colonization of the surfaces of foreign body materials, such as medical devices and implants [121]. Along with more and more implanted medical devices used to improve life quality, the risk of FBAI increases hugely. Additionally, FBAI is difficult to treat since these bacteria embedded in a biofilm are less susceptible to both antibiotics and host defense mechanisms [122]. To develop new anti-biofilm approaches and substances, Fursov et al. determined the efficacy of a broad-range recombinant endolysin LysECD7 against forming and mature biofilms caused by *K. pneumoniae* Ts 141-14 clinical isolate using the implantable diffusion chamber approach. They found that LysECD7 significantly reduced the biofilm formation and was capable of degrading the preformed biofilm in vitro [104]. Recently, a comparative study was conducted to determine the efficacies of endolysin HY-133, daptomycin, and rifampin against *S. aureus* biofilm on the vascular graft surface and found that daptomycin exhibited the strongest bactericidal effect, while HY-133 showed a moderate effect and rifampicin was not effective as an antimicrobial for this biofilm. If considering the risk of resistance, endolysin would be the most favorable antimicrobial agent in this setting [123].

Meanwhile, various staphylococcal endolysins and their derivative proteins are effective at removing biofilms from *S. aureus* and *S. epidermidis*. For example, the staphylococcal endolysins SAP-2 and Phi11 eliminate biofilms formed on polystyrene surfaces [124,125], while the endolysin LysH5 has staphylococcal biofilm-removal properties, with no resistant cells after treatment [126]. Similarly, safranin staining, cell reduction, and scanning electron microscopy have also revealed the effective bacterial removal activities of SAL200 endolysin [127]. PlyGRCS, a staphylococcal endolysin with a single EAD that destroys MRSA, disturbs biofilms as well [128]. The endolysin Lys84 with two catalytic domains (CHAP and amidase_2) and a CBD (SH3b) effectively removed around 90 % of the biofilms of *S. aureus*, and CHAP and Amidase_2 domains remained 61.20 and 59.46 % of lytic activity as well as 84.31 and 70.11 % of the anti-biofilm activity of Lys84, respectively [63]. CHAPk, a truncated LysK endolysin with only the N-terminal endopeptidase domain, can eliminate *S. aureus* biofilms on surfaces [129]. Meng et al. investigated the effect of LySMP, a manufactured phage lysin, on *S. suis* biofilms, both alone and in combination with antibiotics and phages. They reported that LySMP alone could remove >80% of the biofilm, compared to just 20% removal when the biofilm was treated with phages alone and/or antibiotics. Consistent with this, the findings showed that LySMP could treat synergistically *S. suis* biofilm and inactivate the released cells in a concentration-independent manner [130]. A well-known chimeric endolysin ClyR with a concentration of 50 µg/mL was found to remarkably reduce the number of viable cells in 72-h aged *S. mutans* and *S. sobrinus* biofilms after treatment for 5 min. Furthermore, continuous administration of ClyR for 40 days obviously decreased the severity of caries in the rat model infected with a single or a mixed bacteria of *S. mutans* and *S. sobrinus* [131]. The engineered phage endolysin LysRODIΔAmi prevented biofilm formation at low protein concentrations of 0.15–0.6 μM in *S. aureus* and had no toxicity toward human keratinocytes, even at high concentrations of 22.1 μM [132]. 

In addition, endolysins may be more suitable for biofilm eradication than planktonic cells. The combination of chimeric lysins Cpl-711 and PL3 showed an increased synergistic effect on the removal of biofilms compared to planktonic cells in *Streptococcus pneumoniae*. The synergy of Cpl-711 and PL3 was also observed in an adult zebrafish model of pneumococcal infection [133]. In other research, the amidase domain of the *L. monocytogenes* phage vB_LmoS_293 endolysin prevented biofilm formation on abiotic surfaces [94], while the *Salmonella* endolysin Lys68 in combination with malic or citric acid decreased biofilms [134]. The endolysins used for the biofilms formed by *P. aeruginosa* also have been developed. The endolysin LysPA26 resulted in a 1~2 log reduction in biofilm-associated *P. aeruginosa* on a polystyrene plate within 2 h without the use of outer membrane permeabilizers [135]. These findings indicate that endolysins are promising anti-biofilm agents. Nevertheless, the endolysin biofilm-removal abilities should be studied under more accurate conditions, such as flow cell-based models, multispecies biofilm matrixes, and surface coatings or substrates used in food processing facilities [136,137,138,139,140].

## 5. Endolysin Application for Pathogen Detection

The ability to identify pathogens quickly and effectively is critical for disease treatment and prevention. The majority of research has focused on foodborne bacterial detection through phage proteins. In the food industry, the most common pathogens are Gram-positive *L. monocytogenes*, *S. aureus*, and *C. perfringens*, and Gram-negative *Salmonella* and *E. coli.* They cause serious economic losses, foodborne diseases, and death [141,142]. Hence, the food industry requires specific and sensitive diagnostic tools to detect microbial contamination accurately and quickly. Furthermore, the approaches must be both cost-effective and convenient to use [143]. 

Current methods mostly rely on culturing on particular media, or PCR, antibody-based detection, and they are time-consuming and labor-intensive [144]. In addition, the failure of PCR-based methods to distinguish between live and dead cells is a significant drawback. This is important for food diagnostics as PCR will produce positive results even when pathogens have been inactivated, such as through heat treatment. Furthermore, the complex matrix of food can disturb PCR-based detection. Some advanced approaches have been developed for pathogen detection. By utilizing a host recognition protein, H-SA-BP-1, from *S. aureus* phage phiSLT, Idelevich et al. developed a phage-based latex agglutination to detect *S. aureus*, with a specificity of 92.1% and a positive predictive value of 89.6% [145]. A method for rapid detection of bacterial pathogens in blood was also achieved by engineered phages-beads and integrated real-time PCR into MicroChip [146]. Ohlsson et al. integrated acoustic separation, enrichment, and Microchip PCR for detection of bacteria in blood [147].

Interestingly, the use of CBDs for pathogen detection has obvious advantages, such as good maneuverability, lower probability of cross-reactivity, high stability and sensitivity, and rapid detection due to fast target binding [148]. Kretzer et al. proposed a process to recognize *L. monocytogenes* cells on magnetic beads coated with CBDs from various endolysins of *Listeria* phages in 2007, achieving detection rates of >90%, which is better than standard plating technique in terms of both period and sensitivity [143]. Later, Schmelcher and colleagues used this technique to collect *Listeria* cells from inoculated cheese and milk, and they were capable of discriminating serotypes after incubation with CBDs bound with various fluorescent proteins [149]. PlyV12 CBD-functionalized magnetic beads (CBD-MBs) were prepared and used to detect *S. aureus* cells with a detection limit as low as 78 CFU/mL in PBS with less than 50 min, and other bacteria associated common food-borne and nosocomial infections negligibly interfered with this detection, except for *S. epidermidis* [150]. The enhanced green fluorescent protein-fused CBD protein (EGFP-LysCPAS15_CBD1) is able to be used to detect *C. perfringens* within 5 min [84]. Endolysin-based methods for detecting *B. anthracis* have also been developed. Fujinami et al. established a bio-probe to identify *B. anthracis* based on a membrane direct blot assay using the C-terminal region of γ-phage lysin protein (PlyG), which turned out to be simpler and less costly than other genetic tools such as PCR or immunological methods using unique antibodies [151]. Later, Sainathrao et al. showed that a 10-amino-acid motif from the C-terminal region of PlyG, combined with fluorescent Qdot-nanocrystals, is enough to detect *B. anthracis* [152]. Another research group produced an electrochemical impedance sensor by immobilizing the CBD of the endolysin Ply500 from *L. monocytogenes* on a gold sensor surface. In both buffer and artificially contaminated milk, the detection of *Listeria* cells by electrochemical impedance spectroscopy is fast, with detection limits of 10^4^ and 10^5^ CFU/mL, respectively [153]. To detect *S. aureus*, Yu and colleagues developed a CBD-based magnetic enrichment immunoassay [154]. *S. aureus* was captured using immunomagnetic particles (IMPs) coated with IgG antibodies that bind staphylococcal protein A. As such the bacterial cells were concentrated and matrix interference during identification was removed. The second ‘antibody’ in the setup was the biotinylated fusion protein T-CBD of the red fluorescent protein tdTomato and a specific *S. aureus* phage CBD, PlyV12C. Eventually, streptavidin-linked horseradish peroxidase was used to improve the detection sensitivity. In contaminated milk, this setup resulted in a detection limit of 4x10^3^ CFU/ml in 1.5 h [154]. Kong et al. used a surface plasmon resonance (SPR)-chip to incorporate a CBD unique for *B. cereus* phage LysPBC1, which resulted in a detection limit of 10^2^ CFU/mL by using a subtractive inhibition assay [155]. A significant disadvantage of both approaches is that they need advanced and costly equipment [142]. The development of a nitrocellulose-based lateral flow assay (LFA) for the detection of *B. cereus* using the CBD of endolysin LysB4 from *B. cereus* phage B4 was able to avoid this. Briefly, the nitrocellulose membrane is first dipped in a *B. cereus*-containing solution. The bacterial cells adhere to the CBDs that have been immobilized at the membrane test line. In the second step, the membrane is dipped in a solution containing gold nanoparticles, which allows for visualization. The cysteine-glutathione-S-transferase-tagged CBDs (Cys-GSTCBD- AuNP) on these nanoparticles will create a red test line, enabling bacteria to be observed. To summarize, the authors created a CBD-based biosensor that is simple, sensitive, fast, and cost-effective [156]. Furthermore, an exquisite sensitivity was able to be achieved by combining CBD with qPCR, and the bacteria with a limit as low as 2 CFU/mL was detected in the approach [157].

## 6. Endolysin Application in Food Safety

Contamination of foodborne pathogens is a major problem in the food industry. *S. aureus*, *Salmonella* spp., *E. coli*, *L. monocytogenes*, and *Clostridium* spp. contamination can endanger human health, causing financial losses during food processing [46]. It is widely acknowledged that new approaches for reducing pathogenic bacteria in foods are urgently needed. Hence, endolysins have been suggested as a possible alternative biocontrol agent; further, endolysins have already been used to avoid pathogen contamination in food systems [158,159,160,161,162]. For example, endolysin PlyV12 has demonstrated a very high lytic activity against both antibiotic-resistant *E. faecalis* and *E. faecium* [163]. Surprisingly, *L. monocytogenes* can infect plant-based milk. Studies revealed that when LysZ5 is administered to soya milk, it has an excellent sterilization ability [164]. In addition, in the presence of hydrostatic pressure, various alternative phage endolysins such as PlyP825, PlyP40, and Ply511 have successfully treated the pathogen *L. monocytogenes* [165]. In human and animal medicine, *S. aureus* has been identified as a pathogen. Meanwhile, this bacterium is also accountable for food and milk contamination during the manufacturing process [166]. Chang et al. recently confirmed that the presence of a CBD in the staphylococcal endolysin, LysSA11, possesses a key role in specificity and antimicrobial activity as compared to endolysin LysSA97, which showed only moderate activity against *S. aureus* [158].

*Salmonella* is the most common cause of bacterial food poisoning in the United States and many other countries [167]. *Salmonella* disease outbreaks have been found to be related to several foods, including red meats, poultry, fruits, and vegetables [168]. Moreover, several *Salmonella* phage-derived recombinant endolysins have been reported [134,169]. Most of these endolysins have a broad lytic spectrum, particularly when cell membrane-permeabilizing chemicals are jointly used. Lim and colleagues expressed *Salmonella* phage SPN1S endolysin, and it displayed the lytic activity against both *E. coli* and *S.* Typhimurium in a buffer with EDTA to destabilize the cell membranes. Moreover, some activity was also observed for *Shigella*, *Salmonella*, *Pseudomonas*, *Cronobacter*, and *Vibrio* species [170]. In another study, a *Salmonella* phage endolysin Gp110 has a modular structure with an uncharacterized domain of unknown function (DUF3380; pfam11860) in its C terminus, which showed a remarkably high lytic activity against *Salmonella* and other Gram-negative bacteria [169]. Subsequent experimental data have examined the efficacy of endolysins against *B. cereus* as antimicrobials or preservatives for use in the food industry [171,172]. *B. cereus*, a Gram-positive spore-forming bacterium, is responsible for developing both an emetic and a diarrheal toxin which can cause food poisoning [171]. Loessner and colleagues isolated and characterized three different endolysins such as PlyBa, Ply12, and Ply21 from *B. cereus* phage Bastille, TP21, and 12826, respectively. Their effectiveness against Gram-negative and Gram-positive bacteria were measured and all three endolysins were found to be effective against 24 strains of *B. cereus*, as well as many other strains of *B. thuringiensis* [173]. 

Clostridial species are associated with food spoilage in addition to causing diseases in poultry. Germinated *C. sporogenes* and *C. tyrobutyricum* contribute to the development of gases and acids in the dairy industry, which alters the structural and sensory qualities of cheeses [174]. Mayer and friends isolated an N-acetylmuramoyl-L-alanine amidase, CS74, from *C. sporogenes* and documented that the purified protein completely lysed the *C. sporogenes* cells when applied exogenously. The researchers also showed that CS74L was active against *C. acetobutylicum* and *C. tyrobutyricum* using the turbidity assay and fresh bacterial cells, making it a potential cheese bio preservative [175]. Another endolysin isolated from a virulent phage CPT11 was also characterized by the same research group, but this enzyme had a more restricted host range [174]. An endolysin from *C. perfringens*, LysCPAS15, inhibited host cells by up to a 3-log reduction of 2 h [84].

## 7. Endolysin Application in Agriculture

Phytopathogenic bacteria cause many food security problems worldwide [176]. Antibiotics use in agriculture is very controversial because it is unclear how much it contributes to the development of antibiotic resistance in human pathogens [177]. Preferably, an alternative strategy to control phytopathogenic bacteria would be established if its impact is minimal. As a result, endolysins have been suggested as a way to protect plants from bacterial diseases [178]. 

A large number of crops require treatment due to pathogenic infections. Expression of endolysins from phages Atu_ph02 and Atu_ph03 triggered lysis of C58-derived *Agrobacterium tumefaciens*, a Gram-negative soil-borne bacterium [21]. The endolysin PN09 (LysPN09) from *P. syringae* pv. *actinidiae* phage PN09 showed lytic activity against Psa strains. When LysPN09 was coupled with EDTA, Psa strains were effectively damaged, indicating that LysPN09 is a potential candidate for biocontrol of Psa in the kiwifruit industry [48]. The production of transgenic crops that express endolysins to provide defense against pathogenic bacteria is one proposed strategy. After bacterial pectinases break down the plant cells, endolysins accumulate in the tissue and contact with bacteria to inactive them. Transgenic tomato plants with CMP1 phage endolysins were successfully developed two decades ago to prevent infection of *Clavibacter michiganensis*, a bacteria that causes canker [179]. Düring et al. demonstrated the potential of this strategy by growing T4 lysozyme-expressing transgenic potatoes [180]. These genetically modified plants showed resistance to *Pectobacterium carotovora* (formerly *Erwinia carotovora*) species, which cause soft rot [181]. 

A previous study found that by using transgenic plants to design an endolysin-based protection mechanism, it is possible to resolve the antibiotic resistance problem. *Apis* (honey bees) are effective crop pollinators, but they are often infected by *Paenibacillus larvae*, which triggers sepsis and death [166]. Endolysin PlyV12 has high lytic activity against antibiotic-resistant *E. faecalis* and *E. faecium*, which may help to control the emergence of resistance [163]. The bacterium *Xanthomonas oryzae* pv. *oryzae* leads to leaf blight in rice [182] and several antibiotic-resistant strains have been isolated [183]. Lee et al. reported Lys411 from the phage FXo411, which had high lytic activity against *Xanthomonas* [184]. It also showed activity against *Stenotrophomonas maltophilia*, a multidrug-resistant bacterium [184], which is becoming more clinically important in the context of nosocomial infections and immunocompromised patients [185]. However, no follow-up research into Lys411 has been released, and thus the enzyme’s potential for medical or agricultural applications remains unknown. The etiologic agent of crown gall disease in a variety of orchard and vineyard crops is *A. tumefaciens* [186]. Because of its severity and extensive impact, it has been the subject of numerous studies [187]. At present it is generally believed that the lytic protein exhibited intriguing properties, including the ability to not only lyse the cell quickly but also prevent cell division, ensuring potent antimicrobial activity [21]. Therefore, the enzyme is a possible candidate for *A. tumefaciens* biocontrol. However, the mechanisms of implementation must be investigated before a feasible crop protection strategy is established. 

Plants may also be used as bioreactors, not for their safety but for the low-cost, large-scale manufacturing of antimicrobial proteins for human and veterinary medicine. In the chloroplasts of tobacco plants, Oey and colleagues produced the *S. pneumoniae* phage endolysins Cpl-1 and Pal, as well as the group B streptococcal lysin PlyGBS, and the chloroplast-produced protein efficiently inactivated the target bacteria [188,189]. The endolysins LysP11 from *Erysipelothrix rhusiopathiae* produced in *Nicotiana benthamiana* using an *Agrobacterium*-mediated transient expression strategy showed strong antimicrobial activity toward *E. rhusiopathiae* [96]. Similarly, an endolysin-derived triple fusion protein produced in *N. benthamiana* showed growth inhibition against *S. aureus* 305 and Newman [190]. A plant-produced endolysin CP933 was found to inhibit 18% growth of Gram-positive plant pathogenic bacterium *Clavibacter michiganensis* [191]. The practicalities of applying these endolysins on a global scale for individual phytobacteria can be a major challenge, leading to the current lack of knowledge on the use of endolysins for plant bacterial diseases. However, given the high societal cost of plant bacterial diseases when existing treatments fail, the endolysin research should be prioritized. 

**Figure 2:**
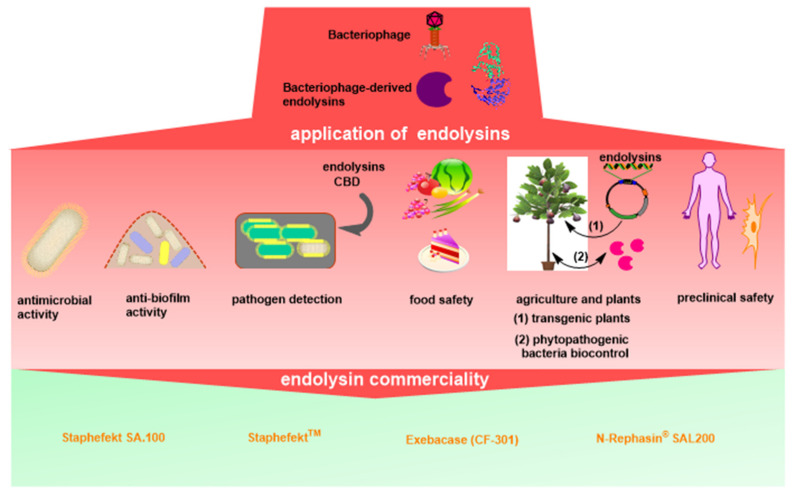
Graphical summary of phage endolysin applications as promising antimicrobial agents.

The different applications of endolysins are summarized in detail in Figure 2.

## 8. Immunogenicity, Toxicity and Safety of Endolysins

Phages are natural components of the human microbiota; therefore, releasing phage-derived endolysins is unlikely to have a detrimental impact on human health [192]. To date, in a variety of animal model systems, the efficacy of phage endolysins has been demonstrated [18,193]. During the preclinical development of protein-based therapeutics such as endolysins, important issues such as safety, toxicity, and immunogenicity must be addressed. Immune responses to foreign proteins, such as the development of anti-drug antibodies, may alter pharmacokinetics, reduce therapeutic effectiveness, and even cause life-threatening complications including hypersensitivity reactions and anaphylaxis. 

Immune responses to well-characterized endolysins such as CF-301 and SAL200 have been identified in a variety of species [166,194]. In phase 1 clinical trial, the protein SAL200 was tested in humans via intravenous infusion. SAL200 is the first MRSA therapeutic formulation based on endolysin. It emerges from the *Staphylococcus* phage SAP-1, which infects staphylococci such as MRSA and vancomycin-resistant *S. aureus* [195]. Healthy male volunteers were given single ascending intravenous doses (0.1 to 10 mg/kg) to test the pharmacokinetics, pharmacodynamics, and tolerance of SAL200 [195]. Volunteers encountered no significant side effects or infection recurrence, with the exception of fatigue, headaches, and myalgia, which were reported by more than three participants. In animal models, the effect of lytic proteins on inflammatory responses or toxicity was investigated, and it was revealed that the administration of certain lysin proteins, such as Cpl-1 and MV-L, caused an immune response that resulted in the development of antibodies against these proteins [196,197]. In another study, endolysin treatment resulted in lower levels of antibodies or cytokine formation in animals compared to untreated controls [198,199]. 

Despite the vast number of published animal trials, only a few endolysins have been tested in humans. Safety analyses with the pneumococcal endolysins Cpl-1 and Pal were performed and it was noticed that IgG levels in mice exposed to these enzymes elevated while IgE levels remained low, implying a low risk of hypersensitivity or allergic reactions. Consequently, no adverse health effects, increased pro-inflammatory cytokine concentrations, or complement activation were observed in mice, confirming that these endolysins have favorable safety and toxicity profiles [199]. 

With the increasing number of protein therapeutics on the market, studies are increasingly focusing on reducing their immunogenicity. The recognition and deletion of T cell epitopes, which can be performed using both experimental and computational methods, is one promising approach in this area. Endolysins may be amenable by similar strategies in future.

## 9. Commerciality of Endolysins

Staphefekt SA.100, an endolysin-based product developed by a Dutch biotech company Micreos, has been available for human use in Europe since 2017. This product is a topical chimeric endolysin that binds to the cell wall of *S. aureus* and cleaves the cell wall using endopeptidase and putative amidase activities [200]. Staphefekt^TM^ improved the clinical symptoms of three human subjects with chronic and recurrent *S. aureus*-related dermatoses in a case study, but they quickly recurred if the therapy was stopped. During chronic and repeated Staphefekt^TM^ therapy, it was also shown that long-term regular use of Staphefekt did not result in the development of bacterial resistance [201]. The application Staphefekt^TM^ on the skin, which targets only *S. aureus* while leaving skin commensals unharmed, improved *S. aureus*-related skin infections including eczema, acne, and rosacea, according to a multicenter, placebo-controlled, double-blinded, and randomized superiority trial study (ClinicalTrials.gov, NCT02840955) [202]. Staphefekt^TM^ is an over-the-counter medication available in the form of a cream or gel that is licensed as a (class 1) medical product in Europe. Several other therapies are in various stages of clinical trials, with some showing promise and paving the way for future endolysin-based therapies [203]. For example, safety and efficacy evaluation of N-Rephasin^®^SAL200 for a single intravenous dose (3 mg/kg) in addition to the conventional standard treatment for the treatment of persistent *S. aureus* bacteremia in patients has been completed in 2019 (phase IIa, NCT03089697). The safety analysis was conducted based on the data of all adverse events, physical examinations, clinical laboratory tests, and vital signs (blood pressure, pulse rate, body temperature, and respiratory rate) collected from the subjects. All subjects who enrolled in this study were defined as the safety set and included in the analysis (https://clinicaltrials.gov/ct2/show/results/NCT03089697?term=SAL-200&draw=2&rank=2) (accessed on 9 October 2021). Another study to evaluate safety, pharmacokinetics, pharmacodynamics, and immunogenicity of N-Rephasin^®^ SAL200 in healthy male volunteers is ongoing (NCT03446053, https://clinicaltrials.gov/ct2/show/results/NCT03446053?term=SAL-200&draw=2&rank=3, accessed on 9 October 2021). Similarly, safety, efficacy, and pharmacokinetics investigation of exebacase (CF-301) for treatment of *S. aureus* bacteremia, including right-sided endocarditis, is ongoing (phase Ⅲ, NCT04160468). The quality control ranges of exebacase were determined as 0.25 to 2 μg/mL and 8 to 64 μg/mL against *S. aureus* ATCC 29213 and *E. faecalis* ATCC 29212, respectively, and were approved by the Clinical and Laboratory Standards Institute (CLSI) [204].

## 10. Conclusions and Perspectives

Since penicillin was discovered, antibiotics have profoundly changed human society and saved lives from deadly bacterial infections. However, the emergence of AMR makes these conventional chemotherapies pale and weak. Phage endolysins are promising weapons against this great challenge of AMR because they exhibit potent and rapid bactericidal and anti-biofilm activity, low induced resistance and cell toxicity, and synergy with regular antibiotics. Remarkably, their narrow antimicrobial spectrums make precise killing possible, no disturbing of the beneficial microbiota. In the meantime, phages are the most abundant and diverse biological entities on the planet. Metagenome studies reveal more and more phages and their endolysin sequences, which is a huge resource of novel endolysins. These sequences also facilitate the combination and assembly of modular domains of chimeric endolysins. In addition, the development of nanomaterial technology and membrane permeabilizer will provide a better delivery strategy for endolysins. Accordingly, endolysins are a new dawn and hope in the dark era of AMR.

## Figures and Tables

**Table 1:** Bacteriophage endolysins of Gram-positive bacteria since 2019.

No.	Endolysin Name	Original Phage	Targeted Pathogens	Effective Concentration	Features of Endolysin	Reference
1	LysMR-5	*S. aureus* phage	*S. aureus*, *S. epidermidis*	500 μg/mL	encapsulation in alginate-chitosan nanoparticles	[59]
2	LysRODI	*S. aureus* phage	*S. aureus*	20 µg/mL	encapsulation in pH-sensitive liposomes, and effective at pH 5	[60]
3	XZ.700	*S. aureus* phage	*S. aureus*	250 µg/mL	chimeric endolysin and effective against *S. aureus* biofilms	[61]
4	LysSAP26	*S. aureus* phage SAP-26	*A. baumannii*, *E. coli*, *K. pneumoniae*, *P. aeruginosa*, *S. aureus*, *E. faecium*	5–80 µg/mL	there was 40% protection rate in *A. baumannii*-infected mouse model	[62]
5	Lys84	*S. aureus* phage qdsa002	*S. aureus*	10 μM	effective against biofilms	[63]
6	LysSAP33	*S. aureus* phage SAP33	*S. aureus*	/	higher activity against biofilms than LysK-like endolysin	[64]
7	S25-3	*S. aureus* kayvirus S25-3	*S. aureus*	/	genus-specific against staphylococci, particularly *S. aureus*	[65]
8	SAL200	*S. aureus* phage	*S. aureus*	/	effective against severe pneumonia caused by *S. aureus* in a lethal murine model	[66]
9	LysCSA13	*S. aureus* phage	*S. aureus*	300 nM	effective against staphylococcal biofilms on various food contact surfaces	[67]
10	Lys109	*S. aureus* phage	*S. aureus*	100 nM	chimeric endolysin	[68]
11	LysP108	*S. aureus* phage	*S. aureus*	/	/	[69]
12	ClyC	*S. aureus* phage	*S. aureus*	/	chimeric endolysin	[70]
13	HY-133	*S. aureus* phage	*S. aureus*	0.12–0.5 μg/mL	chimeric endolysin	[71]
14	LysSA11	staphylococcal phage SA11	*S. aureus*	/	expressed and surface-displayed in *Saccharomyces cerevisiae*	[72]
15	Ph28	*S. epidermidis* phage PH15	*S. epidermidis*	/	/	[73]
16	MSlys	*S. pneumoniae* phage MS1	*S. pneumoniae*	2 μM	/	[74]
17	LyJH307	*Streptococcus bovis* phage	*S. bovis*, *E. faecalis*, *S. sanguinis*	50 µg/mL	highest efficacy at pH 5.5 at 39 °C	[75]
18	LyJH307	*S. bovis*	*S. bovis*	/	as a specific modulator for rumen	[76]
19	PlyC	streptococcal *C1 phage*	group A, C, and E streptococci	/	recognition of *Streptococcus* Group A carbohydrate backbone	[77]
20	LytSD	*S. avermitilis* phage phiSASD1	*S. avermitilis*, *B. subtilis*, *S. aureus*, *S. lutea*, *E. faecalis*	10 μg/mL	/	[78]
21	lys46	*B. subtilis* phage	*K. pneumoniae*, *S.* Typhimurium, *Proteus*, *E. coli*	/	/	[79]
22	Ply57	broad-host-range temperate phage, Izhevsk	*B. cereus* group	1 μM	thermostability at 55 °C	[80]
23	LysPBC5	*B. cereus* phage PBC5	*B. cereus*	/	/	[81]
24	PlyB	*B. anthracis* phage vB_BanS_Bcp1	*B. cereus* sensu lato group species	16 µg/mL	potent bacteriolytic activity against all *B. cereus* sensu lato isolates	[82]
25	LysB4EAD-LysSA11	*B. cereus* phage B4 + *S. aureus* phage SA11	*S. aureus*, *B. cereus*	3.0 µM	a hybrid endolysin	[33]
26	LysPBC2	*B. cereus* phage PBC2	*Bacillus*, *Listeria*, *Clostridium*	/	harboring a *B. cereus* spore binding domain	[30]
27	CTP1L	*C. tyrobutyricum* phage ΦCTP1	*C. tyrobutyricum*	/	the endolysin encoding gene was introduced into the nisin producer *Lactococcus lactis* subsp. *lactis* INIA 415	[83]
28	LysCPAS15	*C. perfringens* phage CPAS-15	*C. perfringens*	45 µg/mL	*C. perfringens*-specific, used for pathogen detection	[84]
29	CWH	*C. difficile* phage phiMMP01	*C. difficile*	/	cell wall binding domain prevents *C. difficile* spore outgrowth	[85]
30	Psa	*C. perfringens* phage st13	*C. perfringens*	/	an amidase endolysin that specifically lyses *C. perfringens*	[86]
31	LysIME-EF1	*E. faecalis* phage	*E. faecalis*	/	a novel two-component endolysin encoded by a single gene	[87]
32	ORF28 endolysin	*E. faecalis* phage ϕEf11	*E. faecalis*	15–31 μg/mL	multifunctional lytic enzyme, effective against *E. faecalis* biofilm	[88]
33	Lys08	*E. faecalis* phage PHB08	*E. faecalis*	0.5–1 µg/mL	effective against *E. faecalis* biofilms	[89]
34	EG-LYS	*E. faecalis* phage	*E. faecalis*	0.1 mg/mL	specific to *E. faecalis*	[90]
35	PBEF129 endolysin	*E. faecalis* phage PBEF129	*E. faecalis*	4.8 µM	effective against biofilm	[91]
36	PM-477	*Gardnerella* prophage	*Gardnerella*	0.13–8 µg/mL	no effect on beneficial lactobacilli or other species of vaginal bacteria	[92]
37	LysKB317	*Lactobacillus* phage EcoSau	*Acetobacter*, *Lactobacillus*, *Pediococcus*, *Streptococcus*, *Weissella*	0.01–1 µM	broad activity and stability from pH 4.5–7.5 up to at least 48 h; maximum activity is observed at 50 °C up to at least 72 h	[93]
38	293 endolysin	*L. monocytogenes* phage vB_LmoS_293	*L. monocytogenes* 473 and 3099, a serotype 4b and serogroup 1/2b-3b-7	/	amidase	[94]
39	LysA	mycobacteriophage D29	*M. smegmatis*	/	separation of *M. smegmatis* from a mixed culture via the cell wall binding domain	[95]
40	LysP11	*Propionibacterium* phage P1.1	*E. rhusiopathiae*	/	binding specifically to the *E. rhusiopathiae* cell wall	[96]
41	PlyPl23	*P. larvae* phage phiIBB_Pl23	*P. larvae*	/	first highly specific CBD targeting exclusively *P. larvae* cells	[97]

Note: “/” indicates data inaccessible.

**Table 2:** Bacteriophage endolysins of Gram-negative bacteria since 2019.

No.	Endolysin Name	Original Phage	Targeted Pathogens	Effective Concentration	Features of Endolysin	Reference
1	LysSS	*S. enterica* serovar Enteritidis phage SS3e	*Salmonella*, *E. coli*, *P. aeruginosa*, *A. baumannii*, *K. pneumoniae*, *S. aureus*	0.063–0.25 mg/mL	/	[98]
2	BSP16Lys	*Salmonella* phage	*S.* Typhimurium, *E. coli*	/	encapsulation into a cationic liposome	[55]
3	LysSE24	*Salmonella* phage LPSE1	*S. enteritidis*	0.1 μM	very stable with different pH (4.0 to 10.0) at different temperatures (20 to 60 °C)	[99]
4	M4Lys	*S. enterica* serovar Typhimurium phage BSPM4	*S. enterica*, *E. coli* O157:H7, *P. aeruginosa*	1 mM	the lysis function was not dependent on either holin or the Sec pathway in vitro	[100]
5	LysSP1	*S.* Typhimurium phage SLMP1	*S.* Typhimurium	50 μg/mL	the optimal activity was at 40 °C and was efficiently active at alkaline condition	[47]
6	LysSTG2	*Salmonella* phage STG2	*Salmonella*, *E. coli*, *P. aeruginosa*	100 μg/mL	effective on *S.* Typhimurium biofilm	[101]
7	LyS15S6	*Salmonella*-virus-FelixO1 phage BPS15S6	3 species of Enterobacteriaceae, *Salmonella*	2 μM	edible ε-poly-L-lysine (EPL) can be used as an outer-membrane permeabilizer	[49]
8	LysECP26	rV5-like phage	*E. coli* O157:H7, *Salmonella* spp.	1 µg/mL	stable at 4–55 °C	[102]
9	Lysep3	*E. coli* phage	*E. coli*	1750 µg/mL	activity was enhanced by modification with hydrophobic amino acids	[51]
10	LysO78	*E. coli* APEC O78 phage vB_EcoM_APEC	*Klebsiella*, *Salmonella*, *Shigella*, *Burkholderia*, *Yersinia*, *Pseudomonas*, *C. arctica*, *E. coli*, *R. solanacearum*, *A. baumannii*	/	the endolysin worked with the help of 50 mM EDTA as membrane permeabilizer	[103]
11	LysECD7	coliphage	*K. pneumoniae*, *Pseudomonas*, *Acinetobacter*	3000 µg/mL	effective against forming and mature biofilm	[104]
12	LysECD7-SMAP	coliphage	*K. pneumoniae*, *Pseudomonas*, *Acinetobacter*	0.5 µg/mL	the endolysin was fused to either the N- or the C-terminus of membrane-destabilizing peptides	[54]
13	Ply6A3	*A. baumannii* phage PD-6A3	*A. baumannii*, *E. coli*, *S. aureus*	1 mg/mL	effective in the mouse sepsis model	[56]
14	Abtn-4	*A. baumannii* phage vB_AbaP_D2	*S. aureus*, *P. aeruginosa*, *K. pneumoniae*, *Enterococcus*, *Salmonella*	5 µM	/	[105]
15	LysAB54	*A. baumannii* phage p54	*A. baumannii*, *P. aeruginosa*, *K. pneumoniae*, *E. coli*	100 μg/mL	/	[106]
16	LysPN09	*P. syringae* pv. *actinidiae* phage PN09	*P. syringae* pv. *actinidiae*	12.5–400 µg/mL	only effective against the outer-membrane-permeabilized Psa strains	[48]
17	RL_Hlys	*P. aeruginosa* phage RL	*P. aeruginosa*, *K. pneumoniae*, *Salmonella*, methicillin resistant *S. aureus*	/	holin was fused at the N terminus of the endolysin	[107]
18	Lysqdvp001	*V. parahaemolyticus* phage	*V. parahaemolyticus*	≥60 U/mL	synergistic effects with ε-poly-lysine	[108]
19	artilysin	*H. pylori* phage KHP30	*H. pylori*	1000 µg/mL	there was a genetic linkage between an endolysin enzyme and a holin enzyme with a section of polypeptides	[53]
20	LysHP1	*H. influenzae* phage HP1	*H. influenzae*, *E. coli*	/	endolysin expression and release was regulated by signal-arrest-release (SAR)	[109]
21	Ts2631	*T. scotoductus* Bacteriophage vB_Tsc2631	the whole Enterobacteriaceae family	1.23 µM	extremely broad antimicrobial activity, especially with EDTA	[58]

Note: “/” indicates data inaccessible.

## Data Availability

Not applicable.
